# Expression of PD-L1 and YWHAZ in Patients with Diffuse Large B Cell Lymphoma: A Possible Association with the Prognosis of Lymphoma

**DOI:** 10.1155/2022/5633096

**Published:** 2022-09-28

**Authors:** Shufang Yan, Qinyu Shang, Zhaopu Fan, Yuping Yang, Yanping Liu, Hongliang Gao, Ken Chen, Fei Liang, Xinxia Li, Qian Zhang, Huifang Yan

**Affiliations:** ^1^Department of Critical Care Medicine of Karamay Central Hospital, No. 67, Junggar Road, Karamay Region, Karamay, Xinjiang Uygur Autonomous Region 834000, China; ^2^Department of Pathology, The Tumor Hospital Affiliated to Xinjiang Medical University, No. 789, Suzhou Dongjie, Urumqi, Xinjiang Uygur Autonomous Region 830011, China; ^3^Xinjiang Medical University, No. 567, North Shangde Road, Urumqi, Xinjiang Uygur Autonomous Region 830011, China; ^4^Department of Gastroenterology, The First Affiliated Hospital of Xinjiang Medical University, No. 137, Liyushan Southern Road, Urumqi, Xinjiang Uygur Autonomous Region 830054, China; ^5^Department of Pathology of Karamay Central Hospital, No. 67, Junggar Road, Karamay Region, Karamay, Xinjiang Uygur Autonomous Region 834000, China

## Abstract

Diffuse large B cell lymphoma (DLBCL) is the most common pathological subtype of non-Hodgkin lymphoma (NHL) and is the most common type of adult lymphoma. Due to the poor prognosis of relapsed/refractory DLBCL, new drug targets and therapeutic methods are urgently needed. We investigated the expression of programmed death ligand 1 (PD-L1) and 3-monooxygenase/tryptophan 5-monooxygenase activating protein zeta (14-3-3*ζ* or YWHAZ) in patients with DLBCL. The purpose was to verify the expression levels of YWHAZ and PD-L1 and their relationships with the prognosis of DLBCL and to lay a foundation for further study on the role of YWHAZ and PD-L1 in DLBCL. Immunohistochemistry was used in 140 patients with DLBCL to test protein expression levels of YWHAZ and PD-L1. All patients were followed up in the hospital or by telephone or via WeChat. The positive expression rate of YWHAZ was 62.14% (87/140). The expression was negatively correlated with the positive expression of BAD (*r* = −0.177, *P* = 0.036) and positively correlated with the positive expression of BCL-2 (*r* = 0.180, *P* = 0.033). When the cut-off value for PD-L1 was established at 5%, 10%, 15%, and 20%, the corresponding positive expression rates of PD-L1 were 79.66% (94/118), 51.69% (61/118), 40.68% (48/118), and 36.44% (43/118). YWHAZ significantly affected the OS of DLBCL (*P* ≤ 0.001). The prognosis of the patients was related to the positive expression of PD-L1 when the cut-off value of PD-L1 was 5% (*P* = 0.033). However, positive expression of PD-L1 was not associated with the prognosis when the cut-off values of PD-L1 were 10% (*P* = 0.404), 15% (*P* = 0.208), and 20% (*P* = 0.408). The positive expression of YWHAZ (hazard ratio 6.215; 95% confidence interval 3.214-12.017; *P* < 0.05) was an independent adverse prognostic factor for OS. YWHAZ may be an important oncogene in the occurrence and development of DLBCL and may be used as a therapeutic target. PD-L1 may be an oncogene or tumor suppressor gene in the occurrence and development of DLBCL. Different cut-off values of PD-L1 may affect the prognosis of DLBCL.

## 1. Introduction

According to the Global Cancer Statistics 2018 [1], the annual growth rate of lymphoma incidence in China is 3%-5%, with approximately 100,000 new cases per year. The age of morbidity tends to be younger. The overall 5-year survival rate of lymphoma patients in China is only 37.2%-38.4% [2, 3], which is much lower than that of the United States (68.1%) and Japan (57.3%). Only 22.1% of patients with diffuse large B cell lymphoma (DLBCL) received more than eight standard treatment courses in China in 2019 [4]. The general poor efficacy and prognosis of lymphoma have become an unbearable burden to patients, individuals, families, and society. Clinical studies have shown that the survival time of lymphoma patients can be significantly prolonged and even cured after standard treatment and comprehensive management [5]. The standardized diagnosis and treatment of lymphoma require cooperation from different disciplines. New therapeutic options, such as monoclonal antibodies, small molecule targeted drugs, and immunotherapy, have significantly improved the short-term efficacy and long-term survival of patients with non-Hodgkin lymphoma (NHL). However, the prognosis of patients with relapsed/refractory (R/R) NHL is still poor, which severely impacts the quality of life. DLBCL is the most common pathological subtype of NHL and the most common type of adult lymphoma. Due to the poor prognosis of R/R DLBCL, new drug targets and therapeutic methods are urgently needed.

In recent years, immune checkpoint inhibitors (ICIs) have been the focus of tumor immunotherapy, and tumor therapy has entered a new era of immunotherapy. Programmed death ligand 1 (PD-L1, also named B7-H1 or CD274), which is overexpressed on tumor cells and tumor-associated macrophages (TAMs), binds to PD-1. PD-1 is expressed in T cells and tumor-infiltrating lymphocytes (TILs), which can inhibit T cell activation via PD-1 during tumor development. T cells appear to function under exhaustion, thus achieving tumor immune escape. Kiyasu et al. [6] show that PD-L1 expression in DLBCL is associated with a poor prognosis. However, McCord et al. [7] obtained the opposite results. Kim et al. [8] suggest that the PD-L1 expression in the primary central nervous system DLBCL (PCNS-DLBCL) is associated with an adverse prognosis. The cut-off values of PD-L1 are controversial. Therefore, the expression level of PD-L1 should be further studied to predict the prognosis of DLBCL.

Our previous study found that the tyrosine 3-monooxygenase/tryptophan 5-monooxygenase activating protein zeta (also named 14-3-3*ζ* or YWHAZ) was differentially expressed according to the isobaric tags for relative and absolute quantification (iTRAQ). Parallel reaction monitoring (PRM) confirmed that YWHAZ was upregulated in DLBCL tissues. The high-level expression of YWHAZ was associated with an unfavorable prognosis of DLBCL [9]. This study was aimed at verifying the expression levels of YWHAZ and PD-L1 and their relationships with the prognosis of DLBCL. The study will lay the foundation for further research on the role of YWHAZ and PD-L1 in DLBCL.

## 2. Material and Methods

### 2.1. Clinical Data

Patient information sex, age, race, and contact information were collected. The following laboratory tests were collected: white blood cell (WBC) count, platelet count, red blood cell (RBC) count, neutrophil granulocyte, monocyte count, hemoglobin, lactate dehydrogenase (LDH), and serum creatinine (Scr). Imaging (CT/MRI/ultrasound) was recorded. Clinical notes included B symptoms (fever, night sweats, and weight loss), first symptoms and locations of the disease, the Ann Arbor Staging classification, Eastern Cooperative Oncology Group performance status (ECOG PS), infectious diseases (HIV, hepatitis B or C, tuberculosis, and syphilis), other systemic diseases (hypertension, diabetes, and heart disease), biopsy (histological/bone marrow), and survival results. The age-adjusted International Prognostic Index (aaIPI) was categorized according to age (≤60 years old and >60 years old). Pathological parameters such as protein expression levels, Ki67 ≥ 70%, and positivity for Epstein-Barr virus (EBV) were also collected.

### 2.2. Pathological Data

#### 2.2.1. Tissue Samples

Informed consent before biopsies was obtained from patients or family members. All samples were obtained from formalin-fixed paraffin-embedded (FFPE) tissues. No treatments were given before this research. The characteristics of all patient samples are recorded in Table 1.

#### 2.2.2. Tissue Microarray and Immunohistochemistry

Tissue chip technology was used for this experiment. One hundred and thirty-seven cases of FFPE tissues were from chips, and three were from three separate slices. The method of testing YWHAZ and PD-L1 was similar to the previous study [10]. However, PD-L1 was detected using an automated immunohistochemical assay. The main antibodies chosen are listed in Table 1. The cut-off values for the overexpression of C-MYC and BCL-2 were ≥40% and ≥70%, respectively. The positive standards for CD10, BCL-6, and MUM-1 were ≥30% [11]. YWHAZ was examined following the manufacturer's protocols. No standard cut-off values of PD-L1 are available. The cut-off values for PD-L1 in this study were tested at ≥5%, ≥10%, ≥15%, and ≥20%, respectively. The diagnostic criteria for double-expressor lymphoma (DEL) or triple-expressor lymphoma (TEL) are the same as in the literature [12]. DLBCL was classified according to the Han's algorithm, consistent with the previous study [12].

### 2.3. In Situ Hybridization

An *in situ* hybridization kit for EBER (EBV-encoded RNA) was purchased from ZSBIO (ISH-5022, ZSBIO, China). The procedure followed the manufacturer's instructions. Nasopharyngeal carcinoma tissues with positive EBER were used as a positive control.

### 2.4. Follow-Up Visits

The first diagnosis was the start of the follow-up visits. Follow-ups ended on July 16, 2021. Follow-up visits were made at the hospital, by telephone, or by WeChat. The reasons for ending follow-up were recorded. Progression-free survival (PFS) is the period between the start of treatment and the observed disease progression or death from any cause. In clinical trials, PFS is often used as a primary or secondary endpoint to determine the effectiveness of a drug in tumor treatment. The survival time was calculated according to the date of the last follow-up visits. The rate of overall survival (OS) is the ratio of the total number of survivors at the end of the follow-up to the total number of recruited patients.

### 2.5. Statistical Analysis

Statistical analysis was conducted using SPSS 23.0, and data were presented using GraphPad Prism 8.0. Unpaired *t*-tests were used to analyze the measurement data, and comparisons were made using the *χ*^2^ test or Fisher's exact test. Cox proportional hazard regression models were used to calculate the hazard ratio (HR) and the 95% confidence interval (95% CI). The Kaplan–Meier method was used to contrast survival rates in univariate analysis.

## 3. Results

### 3.1. Epidemiology and Characteristics of DLBCL Cohort

One hundred forty patients with DLBCL (extranodal 46 and nodal 94) were diagnosed according to histological biopsy in the Department of Pathology from July 2010 to October 2020, and 82 (58.57%) were men. The median age was 59 years, and 64 (45.71%) were ≥60 years old. The study subjects included 91 Han and 49 ethnic minorities. Among the extranodal DLBCL, 32 cases occurred in the central nervous system, 15 in the gastrointestinal tract, and 47 in other body areas. Twenty-eight (20%) cases had Ann Arbor stages І-II, and 112 (80%) had stages III-IV. There were 33 cases with B symptoms. Thirty-one patients (22.14%) had aaIPI > 2. Sixty-eight patients (48.57%) had ECOG PS ≥ 2. There were 66 cases with systemic diseases (12 hepatitis B, 1 Sjögren syndrome, and 53 with other diseases). Thirty-eight cases (27.14%) had positive LDH. Among the 79 patients (56.43%), 2 (2.53%) were EBV positive. One patient had silicosis, and the other had no complications. OS was 5 years and 54 years, and PFS was 0, respectively. Five patients with hemophagocytic syndrome died at the end of the follow-up. Three patients with hemophagocytic syndrome resulted from chemotherapy.

### 3.2. Immunophenotypic Features

#### 3.2.1. The Protein Expression Level Related to Typing and Diagnosis of DLBCL

The expression rates of CD10, BCL-6, CD20, MUM-1, Ki − 67 ≥ 70%, BCL-2, and C-MYC were 22.14%, 83.57%, 100%, 84.29%, 69.29%, 49.29%, and 33.57%, respectively. There were 47 cases of GCB type and 93 cases of non-GCB type. All patients achieved double/triple expression: 19 were positive, including 3 DEL (3/140, 2.14%) and 16 TEL (16/140, 11.43%).

#### 3.2.2. Clinicopathological Features of DLBCL with Different Immunophenotyping

Fifty patients (35.71%, 50/140) were evaluated for bone marrow involvement, and 21 (42%, 21/50) were positive. There were no significant differences in the positive C-MYC expression rate (*P* = 0.768) between patients with GCB and non-GCB subtypes. However, there were significant differences in BCL-6 (*P* = 0.006), MUM-1 (*P* ≤ 0.001), CD10 (*P* ≤ 0.001), and systemic disease (*P* = 0.036). The positive rate of BCL-6 was 77.42% in patients with non-GCB, lower than in patients with the GCB subtype. The positive rate of MUM-1 was 98.92%, higher than in patients with the GCB subtype. The positive CD10 rate was 65.96% in the GCB subtype group, higher than that of the non-GCB subtype group (Table 2).

#### 3.2.3. Immunohistochemical Results

**Figure 1:**
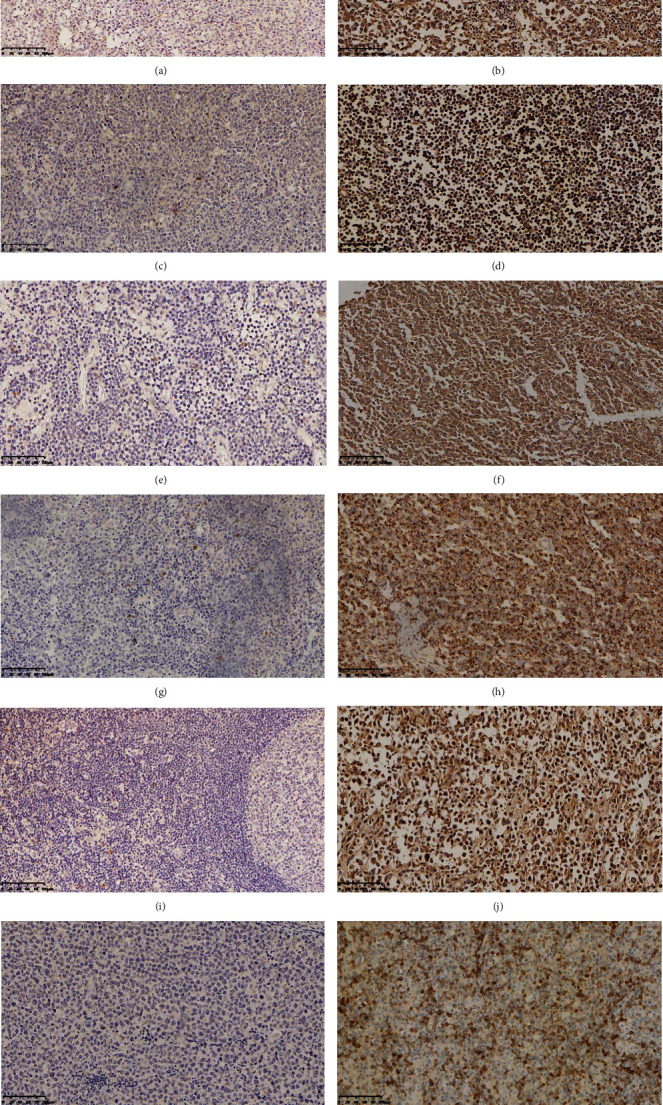
(a~l) Immunohistochemical results (EnVision method, original magnification ∗200) of DLBCL. (a–l) Expressions of YWHAZ, AKT, BAD, BAX, p-AKT, and BCL-2 in DLBCL oncocytes. DLBCL: diffuse large B cell lymphoma. (a, c, e, g, i, k) The expressions of YWHAZ, AKT, BAD, BAX, p-AKT, and BCL-2 were of low-grade positive in the tumor cells, respectively. (b, d, f, h, j, l) The expressions of YWHAZ, AKT, BAD, BAX, p-AKT, and BCL-2 were of high-grade positive in the tumor cells, respectively.

*(1) Expression Levels of Proteins Related to the PI3K/AKT Signal Pathway in DLBCL Tissues*. The protein expression levels of YWHAZ, AKT, BAD, BAX, p-AKT, and BCL-2 detected by immunohistochemistry were 62.14% (87/140, Figures 1(a) and 1(b)), 34.29% (48/140, Figures 1(c) and 1(d)), 29.29% (41/140, Figures 1(e) and 1(f)), 42.14% (59/140, Figures 1(g) and 1(h)), 66.43% (93/140, Figures 1(i) and 1(j)), and 49.29% (69/140, Figures 1(k) and 1(l)), respectively.

*(2) Analysis of the Relationship between YWHAZ and the Expression Levels of Proteins Related to the PI3K/AKT Signaling Pathway, AKT, p-AKT, BAD, BAX, and BCL-2*. The positive expression rate of YWHAZ was 62.14% (87/140). The positive expression of YWHAZ was not correlated with the positive expression of AKT 1 + 2 + 3 (*r* = 0.036, *P* = 0.670), p-AKT (*r* = 0.131, *P* = 0.122), and BAX (*r* = −0.109, *P* = 0.199) in the 140 tissues of DLBCL. The positive expression of YWHAZ was negatively correlated with the positive expressions of BAD (*r* = −0.177, *P* = 0.036) and was positively correlated with BCL-2 (*r* = 0.180, *P* = 0.033).

*(3) Analysis of the Relationship between the Clinicopathological Characteristics of DLBCL and the Expression Levels of Proteins Related to the PI3K/AKT Signaling Pathway-Related YWHAZ, AKT, p-AKT, BAD, BAX, and BCL-2*. The positive expression of YWHAZ was negatively correlated with the positive expression of C-MYC (*r* = −0.225, *P* = 0.008). The expression was positively correlated with low hemoglobin (*r* = 0.243, *P* = 0.004) and ECOG PS ≥ 2 (*r* = 0.228, *P* = 0.007). The positive expression of AKT was positively correlated with age ≥ 60 (*r* = 0.183, *P* = 0.030) and aaIPI > 2 (*r* = 0.195, *P* = 0.021). The positive expression of p-AKT was negatively correlated with B symptoms (*r* = −0.175, *P* = 0.038). The expression was positively correlated with aaIPI > 2 (*r* = 0.197, *P* = 0.020), Ki67 ≥ 70% (*r* = 0.215, *P* = 0.011), and ECOG PS ≥ 2 (*r* = 0.267, *P* = 0.001). The positive expression of BAD was negatively correlated with the positive expression of MUM-1 (*r* = −0.240, *P* = 0.004) and non-GCB subtype (*r* = −0.240, *P* = 0.004). The positive expression of BAX was negatively correlated with the positive expression of MUM-1 (*r* = −0.228, *P* = 0.007), Ki67 ≥ 70% (*r* = −0.184, *P* = 0.029), and non-GCB subtype (*r* = −0.190, *P* = 0.025). The positive expression of BAX was positively correlated with systemic disease (*r* = 0.179, *P* = 0.034) (Table 3).

**Figure 2:**
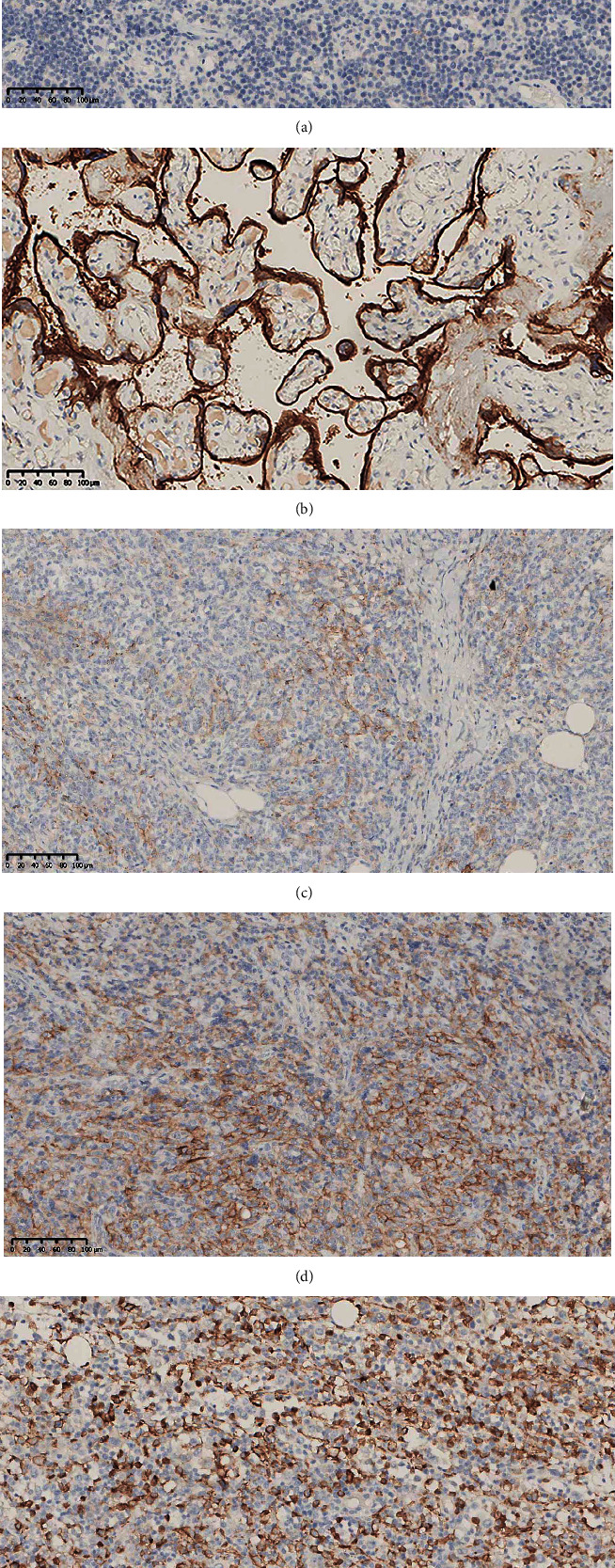
Immunohistochemical results of DLBCL (EnVision method, original magnification 200x) (a–e). (a) Negative control, (b) positive control, (c) PD-L1 weakly positive expression on oncocytes, (d) PD-L1 moderately positive expression on oncocytes, and (e) PD-L1 strong positive expression on oncocytes. DLBCL: diffuse large B cell lymphoma.

*(4) Expression Level of PD-L1 in DLBCL Tissues*. Due to severe tissue detachment, only the FFPE tissues from 118 patients with DLBCL were successfully stained by immunohistochemistry (Figures 2(a)–2(e)). The cut-off values for PD-L1 were set as 5%, 10%, 15%, and 20%, respectively. The corresponding positive expression rates of PD-L1 were 79.66% (94/118), 51.69% (61/118), 40.68% (48/118), and 36.44% (43/118), respectively.

*(5) Analysis of the Relationship between the Clinicopathological Characteristics of DLBCL and the Expression Level of PD-L1 in DLBCL Tissues*. When the cut-off value of PD-L1 was 5%, the positive expression of PD-L1 was negatively correlated with the positive expression of SCr (*r* = −0.230, *P* = 0.012) but positively correlated with B symptoms (*r* = 0.183, *P* = 0.047), C-MYC (*r* = 0.251, *P* = 0.006), and ECOG PS ≥ 2 (*r* = 0.253, *P* = 0.006). When the cut-off value of PD-L1 was 10%, the positive expression of PD-L1 was negatively correlated with the positive expression of BCL-6 (*r* = −0.184, *P* = 0.046) but positively correlated with age ≥ 60 (*r* = 0.205, *P* = 0.026), C-MYC (*r* = 0.288, *P* = 0.002), and ECOG PS ≥ 2 (*r* = 0.187, *P* = 0.043). When the cut-off value of PD-L1 was 15%, the positive expression of PD-L1 was negatively correlated with the positive expression of MUM-1 (*r* = −0.182, *P* = 0.049), monocyte count (*r* = −0.195, *P* = 0.035), and BCL-6 (*r* = −0.201, *P* = 0.029) but positively correlated with C-MYC (*r* = 0.221, *P* = 0.016). When the cut-off value of PD-L1 was 20%, the positive expression of PD-L1 was negatively correlated with the positive expression of BCL-6 (*r* = −0.200, *P* = 0.030) and monocyte count (*r* = −0.224, *P* = 0.015) but positively correlated with C-MYC (*r* = 0.209, *P* = 0.023).

*(6) Follow-Up and Ann Arbor Stage*. Follow-up began at the time of diagnosis with the longest follow-up time of 150 months. Among the 140 patients, 73 died, 127 completed follow-ups, and 13 were lost to follow-ups. The median survival time was 28.5 months (1-150 months). Early death (stages I-II) occurred in 14 cases, and 59 had late death (stages III-IV). There were no significant differences in the percentages of early and late death between DLBCL patients (*X*^2^ = 0.064, *P* = 0.800).

*(7) Survival Analysis*.

1. Univariate survival analysis

**Figure 3:**
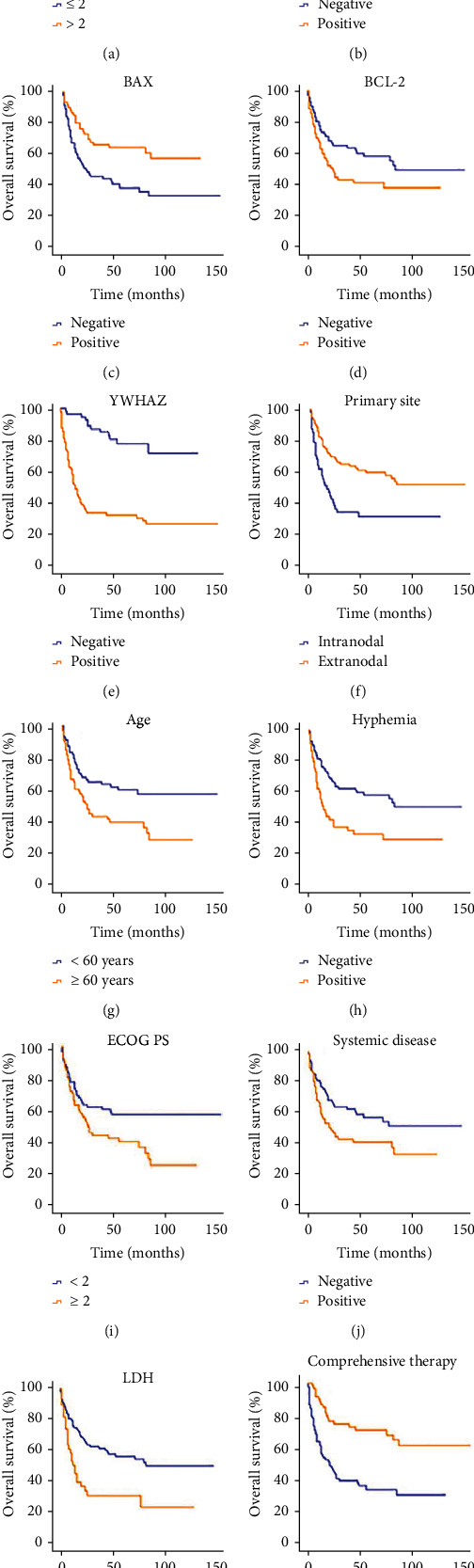
Univariate analysis of patients with DLBCL among various groups. (a–l) Univariate analysis for patients with diffuse large B cell lymphoma among various groups. (a, d–k) The OS of patients with aaIPI > 2, positive BCL-2, positive YWHAZ, primary and nodal site, age ≥ 60, hyphemia, ECOG PS ≥ 2, positive systemic disease, or positive LDH was worse than that of patients with negative ones, respectively. (b, c) The OS of patients with positive BAD or BAX was better than those patients with negative BAD or BAX. (l) The OS of patients receiving comprehensive therapy was longer than those without comprehensive treatment.

Among the 140 patients with DLBCL, aaIPI (*P* = 0.003) (Figure 3(a)), BAD (*P* = 0.048) (Figure 3(b)), BAX (*P* = 0.003) (Figure 3(c)), BCL-2 (*P* = 0.046) (Figure 3(d)), YWHAZ (*P* ≤ 0.001) (Figure 3(e)), primary site (*P* ≤ 0.001) (Figure 3(f)), age (*P* = 0.004) (Figure 3(g)), hyphemia (*P* = 0.002) (Figure 3(h)), ECOG PS (*P* = 0.016) (Figure 3(i)), systemic disease (*P* = 0.024) (Figure 3(j)), LDH (*P* = 0.001) (Figure 3(k)), and treatment mode (*P* ≤ 0.001) (Figure 3(l)) affected the OS. Sex (*P* = 0.806), immunophenotyping (*P* = 0.111), Ann Arbor stage (*P* = 0.995), B symptoms (*P* = 0.522), Ki67 ≥ 70% (*P* = 0.664), p-AKT (*P* = 0.271), AKT (*P* = 0.463), double expression (*P* = 0.301), and triple expression (*P* = 0.692) had no effect on OS.

**Figure 4:**
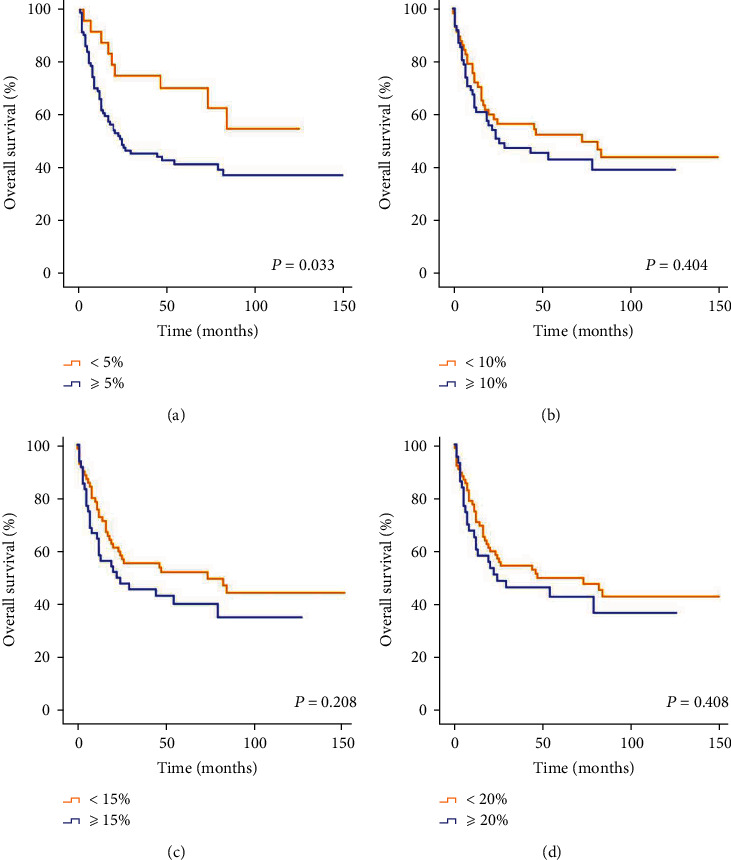
Univariate analysis of patients with diffuse large B cell lymphoma among various groups. (a) The cut-off value of PD-L1 was 5%. (b) The cut-off value of PD-L1 was 10%. (c) The cut-off value of PD-L1 was 15%. (d) The cut-off value of PD-L1 was 20%.

The prognosis of the patients was related to the positive expression of PD-L1 when the cut-off value of PD-L1 was 5% (*P* = 0.033) (Figure 4(a)). However, the positive expression of PD-L1 was not related to the prognosis when the cut-off values of PD-L1 were 10% (*P* = 0.404) (Figure 4(b)), 15% (*P* = 0.208) (Figure 4(c)), and 20% (*P* = 0.408) (Figure 4(d)).

(2) COX multivariate survival analysis

Combined therapy was more effective than surgery alone or chemotherapy alone. The risk of death was lower than that of surgery alone or chemotherapy alone (HR = 0.436; 95% CI, 0.247-0.772; *P* < 0.05). aaIPI > 2 (HR = 2.185; 95% CI, 1.275-3.745; *P* < 0.05), combined systemic disease (HR = 1.706; 95% CI, 1.050-2.772; *P* < 0.05), increased LDH (HR = 1.958; 95% CI, 1.149-3.335; *P* < 0.05), and positive expression of YWHAZ (HR = 6.215; 95% CI, 3.214-12.017; *P* < 0.05) were independent adverse prognostic factors of OS. The positive expression of BAX was an independent protective prognostic factors of OS (HR = 0.478; 95% CI, 0.282-0.810; *P* < 0.05) (Table 4).

Regardless of the cut-off value for PD-L1, age ≥ 60 (HR = 1.915; 95% CI, 1.156-3.174; *P* < 0.05), hyphemia (HR = 1.836; 95% CI, 1.112-3.032; *P* < 0.05), and ECOG PS ≥ 2 (HR = 2.003; 95% CI, 1.207-3.325; *P* < 0.05) were independent adverse prognostic factors of OS. However, PD-L1 was not related to the prognosis.

## 4. Discussion

DLBCL can occur at any age. Most of the patients are middle-aged and older adults. Extranodal diseases account for about 30%-40%, consistent with this study [13, 14]. The prognosis of patients with Ann Arbor staging in the early stage was not different from that in the advanced stage, consistent with a previous study [15]. Because some patients with an advanced stage had been treated in other hospitals before coming to this hospital for treatment, the survival time was longer. The Ann Arbor stage was not related to the prognosis of patients with DLBCL.

In recent years, the morbidity and mortality of primary extranodal DLBCL have risen. Annual OS is also gradually increasing, closely related to the continuous improvement of the lymphoma evaluation system, increased patient awareness of the disease, the development of genetics and molecular biology, and the use of rituximab [16]. The onset of extranodal DLBCL first appears in the central nervous system, followed by the gastrointestinal tract, possibly due to information bias and recall bias. Finally, the prognosis for patients with extranodal DLBCL is better than that for intranodal patients, which is inconsistent with a previous report [17]. The different locations of the primary extranodal DLBCL reflect different clinical features and prognostic effects. Therefore, a new risk stratification feature that involves the origin of the disease is needed to guide the treatment of extranodal DLBCL [18].

Five cases were complicated with the hemophagocytic syndrome and died at the end of follow-up. Among these patients, three were caused by chemotherapy. These patients were critically ill with a poor prognosis and high mortality rate. Clinicians and pathologists should pay enough attention to this type of patient. Many NHL patients have a history of autoimmune diseases, such as Sjögren syndrome, rheumatoid arthritis, or Helicobacter pylori gastritis [19]. Klein et al. [20] hypothesized that continuous disease activity and immune stimulation were the most vital factors for developing DLBCL in patients with rheumatoid arthritis. Consistent with the report [21], we found that DLBCL was a disease diagnosed after Sjögren syndrome.

Compared to sporadic cases, patients with an impaired immune system are more likely to develop EBV-positive DLBCL [22]. This study had two cases of EBV-positive DLBCL: one had silicosis, and the other was diagnosed without complications. The findings are consistent with a previous report [22]. A meta-analysis showed that OS and PFS of EBV-positive DLBCL were significantly worse [23]. These patients should receive a complete course of treatment, especially those with respiratory diseases. There was an autoimmune disorder in patients with silicosis, but no correlation was found [24]. The two cases of EBV-positive DLBCL in this study were sporadic. Because the IPI score is related to performance status, it is difficult to determine whether poor performance status is caused by rheumatic disease or DLBCL in patients with DLBCL with rheumatic diseases.

The prognosis of patients with DLBCL is closely related to hepatitis B virus infection and replication [25, 26]. In this study, due to the small number of samples and cases of hepatitis B combined with other chronic diseases, it is impossible to accurately assess its effect on the prognosis of DLBCL. Therefore, more research is needed. At the same time, paying more attention to the patients with DLBCL complicated by hepatitis B infection is necessary. Clinicians should pay attention to patients with systemic diseases, especially hepatitis B and Sjögren syndrome. It is essential to follow these patients closely. The level of virus replication before chemotherapy should be assessed to avoid reactivating the virus, causing virus DNA replication, increasing the difficulty of treatment, and accelerating patient death.

A daily examination of bone marrow involvement should be performed to adjust the treatment scheme. The Ki67 index of the non-GCB subtype is higher than that of the GCB subtype, which contradicted the results of this study [27]. Some studies showed a reverse relationship between the Ki67 index and clinical prognosis, while others did not [28]. The importance of early immunohistochemical and FISH detection in predicting the prognosis of DLBCL should be noted. Under standard treatment, patients with the non-GCB subtype tend to have a poorer prognosis than those with the GCB type [29], which is confirmed in this study. Ki67 is associated with the prognosis of patients with primary intestinal DLBCL [30], unlike the results in this study. The findings may be related to the range of Ki67 cut-off values and the selection of research objects.

The positive expression of YWHAZ was correlated with BAD and BCL-2, suggesting that YWHAZ may be related to apoptosis and YWHAZ may restore sensitivity to CHOP-induced apoptosis [31]. The protein expression level of YWHAZ was not correlated with those of p-AKT and AKT. This could be due to the low positive rates of AKT and p-AKT proteins in immunohistochemistry, which were affected by various factors, such as the preservation time of the selected samples, antibodies without high sensitivity, and the technique of the experimenter. These factors could lead to false negative results. The correlation can be further analyzed by expanding the sample size or performing experiments using Western blot or qRT-PCR methods. Positive expressions of the YWHAZ, AKT, and p-AKT proteins might promote the development and proliferation of DLBCL, related to the poor prognosis of DLBCL. BAD and BAX were associated with a good prognosis of DLBCL in this study. BAX might be related to DLBCL proliferation and inhibiting tumor cell proliferation. The prognosis can be preliminarily evaluated according to the expression level of BAD. Consistent with the previous report, the positive expression of YWHAZ is related to the prognosis of DLBCL [9] with the short OS. It is suggested that YWHAZ may be an oncogene in DLBCL, which is worthy of further study.

Although the cut-off value of PD-L1 was different, C-MYC was consistently positively correlated with the positive expression of PD-L1 in our research. The positive expression of C-MYC protein in DLBCL is related to poor prognosis [32], suggesting that PD-L1 may be an oncogene in DLBCL. When the cut-off value of PD-L1 was 10%, 15%, and 20%, BCL-6 was always negatively correlated with prognosis. However, BCL-6 was found to be an oncogene in DLBCL [33]. PD-L1 may be a tumor suppressor gene in DLBCL. It can be seen that PD-L1 with different cut-off values has different effects on the prognosis of DLBCL. When the cut-off value of PD-L1 was 5%, the positive expression of PD-L1 was related to the poor prognosis of DLBCL. The result is consistent with some reports [6, 8] but inconsistent with this report [7]. The expression of PD-L1 is significantly correlated with the prognosis of melanoma patients when 5% is used as the cut-off value, which is consistent with this study [34]. PD-L1 may be an oncogene or tumor suppressor gene in DLBCL, which provides a new target for the treatment of R/R DLBCL. Macrophages are the main source of PD-L1 expression in the tumor microenvironment (TME) of DLBCL [35]. Based on this study, our team intends to explore the relationship between the expression of TAM and PD-L1 expression in TME and their effects on the prognosis of DLBCL.

In this study, the OS of patients with DLBCL treated with comprehensive treatment was higher than with simple surgical resection or chemotherapy, and the prognosis was better. Therefore, clinicians should first recommend comprehensive treatment when formulating the treatment plan to improve OS and quality of life. We also found that the ECOG score, hyphemia, complicated systemic diseases (such as hepatitis B and Sjögren syndrome), LDH, aaIPI, and BCL-2 were related to the prognosis of DLBCL. However, sex was not associated with the prognosis of DLBCL. These results were consistent with some studies [36–40] but inconsistent with others [37, 38, 41].

## 5. Conclusions

YWHAZ may be an important oncogene in the occurrence and development of DLBCL and may be used as a therapeutic target. PD-L1 may be an oncogene or tumor suppressor gene in the occurrence and development of DLBCL. Different cut-off values of PD-L1 may have different effects on the prognosis of DLBCL. These findings may suggest novel targets for the diagnosis and therapy of DLBCL.

## Figures and Tables

**Table 1:** Primary antibodies and conditions used for immunohistochemical staining.

Antigen	Clone	Source	Dilution	Positive location
YWHAZ	ab51129	Abcam	1 : 100	Cytomembrane/cytoplasm
PD-L1	SP263	Roche	Ready to use	Cytomembrane/cytoplasm
BCL-2	100/D5	Shanghai Long Island	1 : 100	Cytoplasm
BCL-6	GI191E/A8	ZSBIO	1 : 80	Nuclei
AKT1+2+3	ab38449	Abcam	1 : 100	Cytomembrane/cytoplasm/nuclei
p-AKT	ab8805	Abcam	1 : 100	Cytomembrane/cytoplasm/nuclei
BAD	ab32445	Abcam	1 : 1000	Cytomembrane/cytoplasm
BAX	ab32503	Abcam	1 : 500	Cytoplasm
MUM-1	EPR5653	Abcam	1 : 200	Nuclei
C-MYC	Y69	ZSBIO	1 : 150	Nuclei
Ki-67	MIB-1	Gene company	1 : 150	Nuclei
CD10	56C6	Gene company	1 : 50	Cytomembrane
CD20	L26	Dako	1 : 150	Cytomembrane

**Table 2:** The clinicopathological features of 140 DLBCL patients.

Item	GCB group	Non-GCB group	*P* value
*n*	47	93	—
Age (year)			
Mean ± SD	54.53 ± 12.97	58.43 ± 15.36	0.283
Sex, *n* (%)			
Male	29 (61.7)	53 (57.0)	0.593
Female	18 (38.3)	40 (43.0)	
Primary localization, *n* (%)			
Extranodal	30 (63.8)	64 (68.8)	0.553
Nodal	17 (36.2)	29 (31.2)	
C-MYC, *n* (%)			
Positive	15 (31.9)	32 (34.4)	0.768
Negative	32 (68.1)	61 (65.6)	
BCL-6, *n* (%)			
Positive	45 (95.7)	72 (77.4)	0.006^∗^
Negative	2 (4.3)	21 (22.6)	
MUM-1, *n* (%)			
Positive	26 (55.3)	92 (98.9)	≤0.001
Negative	21 (44.7)	1 (1.1)	
CD10, *n* (%)			
Positive	31 (66.0)	0 (0)	≤0.001
Negative	16 (34.0)	93 (100)	
LDH, *n* (%)			
Positive	12 (25.5)	26 (28.0)	0.761
Negative	35 (74.5)	67 (72.0)	
SCr, *n* (%)			
Positive	1 (2.1)	5 (5.4)	0.664
Negative	46 (97.9)	88 (94.6)	
Monocyte count (10^9^/L), *n* (%)			
Decreased or increased	12 (25.5)	32 (34.4)	0.285
Normal	35 (74.5)	61 (65.6)	
RBC (10^9^/L), *n* (%)			
Decreased or increased	27 (57.4)	58 (62.4)	0.574
Normal	20 (42.6)	35 (37.6)	
Hemoglobin (g/L), *n* (%)			
Decreased or increased	13 (27.7)	31 (33.3)	0.495
Normal	34 (72.3)	62 (66.7)	
Platelet count (10^9^/L), *n* (%)			
Decreased or increased	13 (27.7)	20 (21.5)	0.418
Normal	34 (72.3)	73 (78.5)	
WBC (10^9^/l), *n* (%)			
Decreased or increased	10 (21.3)	20 (21.5)	0.975
Normal	37 (78.7)	73 (78.5)	
LDH, *n* (%)			
Positive	28 (59.6)	38 (40.9)	0.036^∗^
Negative	19 (40.4)	55 (59.1)	
Ki67, *n* (%)			
≥70%	34 (72.3)	63 (67.7)	0.578
<70%	13 (27.7)	30 (32.3)	
ECOG PS, *n* (%)			
≥2	18 (38.3)	50 (53.8)	0.084
<2	29 (61.7)	43 (46.2)	
Ann Arbor stage, *n* (%)			
I-II (early stage)	11 (23.4)	17 (18.3)	0.474
III-IV (late stage)	36 (76.6)	76 (81.7)	
B symptoms, *n* (%)			
Positive	7 (14.9)	26 (28.0)	0.085
Negative	40 (85.1)	67 (72.0)	
aaIPI, *n* (%)			
>2	6 (12.8)	25 (26.9)	0.057
≤2	41 (87.2)	68 (73.1)	
Treatment options, *n* (%)			
Comprehensive treatment	20 (42.6)	39 (41.9)	0.944
Operation or chemotherapy	27 (57.4)	54 (58.1)	

^∗^ *P* < 0.05 represents statistical differences. DLBCL: diffuse large B cell lymphoma; LDH: lactate dehydrogenase; SCr: serum creatinine; WBC: white blood cell count; RBC: red blood cell count; GCB subtype: germinal center B cell-like subtype; Non-GCB subtype: nongerminal center B cell-like subtype; ECOG PS: Eastern Cooperative Oncology Group performance status.

**Table 3:** Relationship between the protein expression levels of YWHAZ, AKT, p-AKT, BAD, BAX, and BCL-2 and clinicopathological features in patients with DLBCL (*n* = 140).

Item	YWHAZ		AKT		p-AKT		BAD		BAX		BCL-2	
	Positive	Negative	Positive	Negative	Positive	Negative	Positive	Negative	Positive	Negative	Positive	Negative
Age (year), *n* (%)												
≥60	42 (48.3%)	22 (41.5%)	28 (58.3%)	36 (39.1%)	43 (46.2%)	21 (44.7%)	14 (34.1%)	50 (50.5%)	29 (49.2%)	35 (43.2%)	32 (46.4%)	32 (45.1%)
<60	45 (51.7%)	31 (58.5%)	20 (41.7%)	56 (60.9%)	50 (53.8%)	26 (55.3%)	27 (65.9%)	49 (49.5%)	30 (50.8%)	46 (56.8%)	37 (53.6%)	39 (54.9%)
*P* value	0.439		0.030^∗^		0.863		0.078		0.489		0.314	

Sex, *n* (%)												
Male	50 (57.5%)	32 (60.4%)	28 (58.3%)	54 (58.7%)	53 (57.0%)	29 (61.7%)	25 (61.0%)	57 (57.6%)	32 (54.2%)	50 (61.7%)	37 (53.6%)	45 (63.4%)
Female	37 (42.5%)	21 (39.6%)	20 (41.7%)	38 (41.3%)	40 (43.0%)	18 (38.3%)	16 (39.0%)	42 (42.4%)	27 (45.8%)	31 (38.3%)	32 (46.4%)	26 (36.6%)
*P* value	0.737		0.967		0.596		0.713		0.378		0.244	

C-MYC, *n* (%)												
Positive	22 (25.3%)	25 (47.2%)	11 (22.9%)	36 (39.1%)	27 (29.0%)	20 (42.6%)	17 (41.5%)	30 (30.3%)	18 (30.5%)	29 (35.8%)	20 (29.0%)	27 (38.0%)
Negative	65 (74.7%)	28 (52.8%)	37 (77.1%)	56 (60.9%)	66 (71.0%)	27 (57.4%)	24 (58.5%)	69 (69.7%)	41 (69.5%)	52 (64.2%)	49 (71.0%)	44 (62.0%)
*P* value	0.008^∗^		0.054		0.111		0.206		0.516		0.261	

BCL-6, *n* (%)												
Positive	70 (80.5%)	47 (88.7%)	40 (83.3%)	77 (83.7%)	77 (82.8%)	40 (85.1%)	37 (90.2%)	80 (80.8%)	52 (88.1%)	65 (80.2%)	58 (84.1%)	59 (83.1%)
Negative	17 (19.5%)	6 (11.3%)	8 (16.7%)	15 (16.3%)	16 (17.2%)	7 (14.9%)	4 (9.8%)	19 (19.2%)	7 (11.9%)	16 (19.8%)	11 (15.9%)	12 (16.9%)
*P* value	0.206		0.957		0.730		0.173		0.216		0.879	

MUM-1, *n* (%)												
Positive	75 (86.2%)	43 (81.1%)	41 (85.4%)	77 (83.7%)	82 (88.2%)	36 (76.6%)	29 (70.7%)	89 (89.9%)	44 (74.6%)	74 (91.4%)	57 (82.6%)	61 (85.9%)
Negative	12 (13.8%)	10 (18.9%)	7 (14.6%)	15 (16.3%)	11 (11.8%)	11 (23.4%)	12 (29.3%)	10 (10.1%)	15 (25.4%)	7 (8.6%)	12 (17.4%)	10 (14.1%)
*P* value	0.427		0.792		0.076		0.004^∗^		0.007^∗^		0.594	

CD10, *n* (%)												
Positive	16 (18.4%)	15 (28.3%)	13 (27.1%)	18 (19.6%)	20 (21.5%)	11 (23.4%)	13 (31.7%)	18 (18.2%)	15 (25.4%)	16 (19.8%)	18 (26.1%)	13 (18.3%)
Negative	71 (81.6%)	38 (71.7%)	35 (72.9%)	74 (80.4%)	73 (78.5%)	36 (76.6%)	28 (68.3%)	81 (81.8%)	44 (74.6%)	65 (80.2%)	51 (73.9%)	58 (81.7%)
*P* value	0.173		0.313		0.800		0.080		0.429		0.271	

LDH, *n* (%)												
Positive	27 (31.0%)	11 (20.8%)	14 (29.2%)	24 (26.1%)	28 (30.1%)	10 (21.3%)	14 (34.1%)	24 (24.2%)	17 (28.8%)	21 (25.9%)	19 (27.5%)	19 (26.8%)
Negative	60 (69.0%)	42 (79.2%)	34 (70.8%)	68 (73.9%)	65 (69.9%)	37 (78.7%)	27 (65.9%)	75 (75.8%)	42 (71.2%)	60 (74.1%)	50 (72.5%)	52 (73.2%)
*P* value	0.187		0.700		0.270		0.233		0.707		0.919	

SCr, *n* (%)												
Increased	2 (2.3%)	4 (7.5%)	2 (4.2%)	4 (4.3%)	6 (6.5%)	0 (0.0%)	1 (2.4%)	5 (5.1%)	1 (1.7%)	5 (6.2%)	3 (4.3%)	3 (4.2%)
Normal	85 (97.7%)	49 (92.5%)	46 (95.8%)	88 (95.7%)	87 (93.5%)	47 (100.0%)	40 (97.6%)	94 (94.9%)	58 (98.3%)	76 (93.8%)	66 (95.7%)	68 (95.8%)
*P* value	0.139		0.960		0.076		0.491		0.199		0.972	
Immunophenotyping, *n* (%)												
Non-GCB	62 (71.3%)	31 (58.5%)	32 (66.7%)	61 (66.3%)	66 (71.0%)	27 (57.4%)	20 (48.8%)	73 (73.7%)	33 (55.9%)	60 (74.1%)	43 (62.3%)	50 (70.4%)
GCB	25 (28.7%)	22 (41.5%)	16 (33.3%)	31 (33.7%)	27 (29.0%)	20 (42.6%)	21 (51.2%)	26 (26.3%)	26 (44.1%)	21 (25.9%)	26 (37.7%)	21 (29.6%)
*P* value	0.122		0.966		0.111		0.004^∗^		0.025^∗^		0.878	

Ann Arbor stage, *n* (%)												
III-IV (late stage)	74 (85.1%)	38 (71.7%)	41 (85.4%)	71 (77.2%)	76 (81.7%)	36 (76.6%)	33 (80.5%)	79 (79.8%)	44 (74.6%)	68 (84.0%)	54 (78.3%)	58 (81.7%)
I-II (early stage)	13 (14.9%)	15 (28.3%)	7 (14.6%)	21 (22.8%)	17 (18.3%)	11 (23.4%)	8 (19.5%)	20 (20.2%)	15 (25.4%)	13 (16.0%)	15 (21.7%)	13 (18.3%)
*P* value	0.056		0.250		0.478		0.927		0.173		0.615	

Monocyte count (10^9^/l), *n* (%)												
Decreased or increased	31 (35.6%)	13 (24.5%)	10 (20.8%)	34 (37.0%)	31 (33.3%)	13 (27.7%)	13 (31.7%)	31 (31.3%)	17 (28.8%)	27 (33.3%)	22 (31.9%)	22 (31.0%)
Normal	56 (64.4%)	40 (75.5%)	38 (79.2%)	58 (63.0%)	62 (66.7%)	34 (72.3%)	28 (68.3%)	68 (68.7%)	42 (71.2%)	54 (66.7%)	47 (68.1%)	49 (69.0%)
*P* value	0.172		0.052		0.498		0.964		0.573		0.910	

B symptoms, *n* (%)												
Positive	23 (26.4%)	10 (18.9%)	7 (14.6%)	26 (28.3%)	17 (18.3%)	16 (34.0%)	7 (17.1%)	26 (26.3%)	11 (18.6%)	22 (27.2%)	14 (20.3%)	19 (26.8%)
Negative	64 (73.6%)	43 (81.1%)	41 (85.4%)	66 (71.7%)	76 (81.7%)	31 (66.0%)	34 (82.9%)	73 (73.7%)	48 (81.4%)	59 (72.8%)	55 (79.7%)	52 (73.2%)
*P* value	0.310		0.071		0.038^∗^		0.247		0.244		0.371	

AaIPI, *n* (%)												
>2	22 (25.3%)	9 (17.0%)	16 (33.3%)	15 (16.3%)	26 (28.0%)	5 (10.6%)	8 (19.5%)	23 (23.2%)	13 (22.0%)	18 (22.2%)	20 (29.0%)	11 (15.5%)
≤2	65 (74.7%)	44 (83.0%)	32 (66.7%)	77 (83.7%)	67 (72.0%)	42 (89.4%)	33 (80.5%)	76 (76.8%)	46 (78.0%)	63 (77.8%)	49 (71.0%)	60 (84.5%)
*P* value	0.254		0.021^∗^		0.020^∗^		0.632		0.979		0.055	

RBC (10^9^/L), *n* (%)												
Decreased	56 (64.4%)	29 (54.7%)	24 (50.0%)	61 (66.3%)	53 (57.0%)	32 (68.1%)	27 (65.9%)	58 (58.6%)	31 (52.5%)	54 (66.7%)	45 (65.2%)	40 (56.3%)
Normal	31 (35.6%)	24 (45.3%)	24 (50.0%)	31 (33.7%)	40 (43.0%)	15 (31.9%)	14 (34.1%)	41 (41.4%)	28 (47.5%)	27 (33.3%)	24 (34.8%)	31 (43.7%)
*P* value	0.260		0.061		0.207		0.427		0.092		0.285	

Hemoglobin (g/L), *n* (%)												
Decreased	35 (40.2%)	9 (17.0%)	15 (31.3%)	29 (31.5%)	30 (32.3%)	14 (29.8%)	11 (26.8%)	33 (33.3%)	21 (35.6%)	23 (28.4%)	20 (29.0%)	24 (33.8%)
Normal	52 (59.8%)	44 (83.0%)	33 (68.7%)	63 (68.5%)	63 (67.7%)	33 (70.2%)	30 (73.2%)	66 (66.7%)	38 (64.4%)	58 (71.6%)	49 (71.0%)	47 (66.2%)
*P* value	0.004^∗^		0.974		0.768		0.454		0.369		0.543	

Platelet count (10^9^/L), *n* (%)												
Decreased	22 (25.3%)	11 (20.8%)	10 (20.8%)	23 (25.0%)	19 (20.4%)	14 (29.8%)	9 (22.0%)	24 (24.2%)	13 (22.0%)	20 (24.7%)	16 (23.2%)	17 (23.9%)
Normal	65 (74.7%)	42 (79.2%)	38 (79.2%)	69 (75.0%)	74 (79.6%)	33 (70.2%)	32 (78.0%)	75 (75.8%)	46 (78.0%)	61 (75.3%)	53 (76.8%)	54 (76.1%)
*P* value	0.543		0.585		0.221		0.773		0.717		0.917	
WBC (10^9^/L), *n* (%)												
Decreased or increased	19 (21.8%)	11 (20.8%)	10 (20.8%)	20 (21.7%)	19 (20.4%)	11 (23.4%)	9 (22.0%)	21 (21.2%)	9 (15.3%)	21 (25.9%)	15 (21.7%)	15 (21.1%)
Normal	68 (78.2%)	42 (79.2%)	38 (79.2%)	72 (78.3%)	74 (79.6%)	36 (76.6%)	32 (78.0%)	78 (78.8%)	50 (84.7%)	60 (74.1%)	54 (78.3%)	56 (78.9%)
*P* value	0.881		0.902		0.688		0.923		0.130		0.930	

Systemic diseases, *n* (%)												
Positive	42 (48.3%)	24 (45.3%)	25 (52.1%)	41 (44.6%)	44 (47.3%)	22 (46.8%)	20 (48.8%)	46 (46.5%)	34 (57.6%)	32 (39.5%)	37 (53.6%)	29 (40.8%)
Negative	45 (51.7%)	29 (54.7%)	23 (47.9%)	51 (55.4%)	49 (52.7%)	25 (53.2%)	21 (51.2%)	53 (53.5%)	25 (42.4%)	49 (60.5%)	32 (46.4%)	42 (59.2%)
*P* value	0.733		0.401		0.955		0.804		0.034^∗^		0.132	

Primary localization, *n* (%)												
Extranodal	59 (67.8%)	35 (66.0%)	37 (77.1%)	57 (62.0%)	61 (65.6%)	33 (70.2%)	26 (63.4%)	68 (68.7%)	44 (74.6%)	50 (61.7%)	46 (66.7%)	48 (67.6%)
Nodal	28 (32.2%)	18 (34.0%)	11 (22.9%)	35 (38.0%)	32 (34.4%)	14 (29.8%)	15 (36.6%)	31 (31.3%)	15 (25.4%)	31 (38.3%)	23 (33.3%)	23 (32.4%)
*P* value	0.829		0.071		0.586		0.549		0.112		0.907	

Ki67, *n* (%)												
≥70%	59 (67.8%)	38 (71.7%)	35 (72.9%)	62 (67.4%)	71 (76.3%)	26 (55.3%)	31 (75.6%)	66 (66.7%)	35 (59.3%)	62 (76.5%)	49 (71.0%)	48 (67.6%)
<70%	28 (32.2%)	15 (28.3%)	13 (27.1%)	30 (32.6%)	22 (23.7%)	21 (44.7%)	10 (24.4%)	33 (33.3%)	24 (40.7%)	19 (23.5%)	20 (29.0%)	23 (32.4%)
*P* value	0.632		0.505		0.011^∗^		0.300		0.029^∗^		0.665	

ECOG PS, *n* (%)												
≥2	50 (57.5%)	18 (34.0%)	27 (56.3%)	41 (44.6%)	54 (58.1%)	14 (29.8%)	19 (46.3%)	49 (49.5%)	25 (42.4%)	43 (53.1%)	38 (55.1%)	30 (42.3%)
<2	37 (42.5%)	35 (66.0%)	21 (43.7%)	51 (55.4%)	39 (41.9%)	33 (70.2%)	22 (53.7%)	50 (50.5%)	34 (57.6%)	38 (46.9%)	31 (44.9%)	41 (57.7%)
*P* value	0.007^∗^		0.192		0.001^∗^		0.736		0.213		0.131	

The calculation of *P* value is related to the Spearman rank. ^∗^*P* < 0.05 represents statistical differences. DLBCL: diffuse large B cell lymphoma; LDH: lactate dehydrogenase; Scr: serum creatinine; WBC: white blood cell count; RBC: red blood cell count; GCB subtype: germinal center B cell-like subtype; Non-GCB subtype: nongerminal center B cell-like subtype; ECOG PS: Eastern Cooperative Oncology Group performance status.

**Table 4:** Multivariate Cox regression analysis.

Influencing factors	B	Standard error	Wald	df	Prominence	Exp(B)	Exp(B) 95% confidence interval	
							Minimum	Maximum
Treatment options	-0.829	0.291	8.019	1	0.004	0.436	0.247	0.772
aaIPI > 2	0.782	0.275	8.092	1	0.004	2.185	1.275	3.745
Combined systemic disease	0.534	0.248	4.651	1	0.031	1.706	1.050	2.772
LDH	0.672	0.272	6.108	1	0.013	1.958	1.149	3.335
YWHAZ	1.827	0.336	29.484	1	≤0.001	6.215	3.214	12.017
BAX	-0.739	0.269	7.527	1	0.006	0.478	0.282	0.810

## Data Availability

All data generated or analyzed during this study are included in this published article.
